# Moderate alcohol consumption is associated with better endothelial function: a cross sectional study

**DOI:** 10.1186/1471-2261-9-8

**Published:** 2009-02-20

**Authors:** Keiko Suzuki, Mitchell SV Elkind, Bernadette Boden-Albala, Zhezhen Jin, Grace Berry, Marco R Di Tullio, Ralph L Sacco, Shunichi Homma

**Affiliations:** 1 Division of Cardiology, Department of Medicine, Columbia University College of Physicians and Surgeons, 630 West 168th Street, New York, NY, USA; 2 Department of Neurology, Columbia University College of Physicians and Surgeons and Mailman School of Public Health, 630 West 168th Street, New York, NY, USA; 3 Department of Sociomedical Science, Mailman School of Public Health, Columbia University College of Physicians and Surgeons, 630 West 168th Street, New York, NY, USA; 4 Department of Biostatistics, Mailman School of Public Health, Columbia University College of Physicians and Surgeons, 722 West 168th Street, New York, NY, USA; 5 Department of Neurology, University of Miami, Miller School of Medicine, 1120 Northwest 14th Street, Miami, FL, USA

## Abstract

**Background:**

Moderate alcohol consumption is protective against coronary artery disease. Endothelial dysfunction contributes to atherosclerosis and the pathogenesis of cardiovascular disease. The effects of alcohol consumption on endothelial function may be relevant to these cardiovascular outcomes, but very few studies have examined the effect of alcohol consumption on endothelial function assessed by flow-mediated dilation (FMD) of the brachial artery in humans.

**Methods:**

In the population-based Northern Manhattan Study (NOMAS), we performed a cross-sectional analysis of lifetime alcohol intake and brachial artery FMD during reactive hyperemia using high-resolution B-mode ultrasound images among 884 stroke-free participants (mean age 66.8 years, women 56.6%, Hispanic 67.4%, black 17.4%, and white 15.2%).

**Results:**

The mean brachial FMD was 5.7% and the median was 5.5%. Compared to non-drinkers, those who drank >1 drink/month to 2 drinks/day were more likely to have FMD above the median FMD (5.5%) (unadjusted OR 1.7, 95% CI 1.2–2.4, p = 0.005). In multivariate analysis, the relationship between moderate alcohol consumption and FMD remained significant after adjusting for multiple traditional cardiovascular risk factors, including sex, race-ethnicity, body mass index, diabetes mellitus, coronary artery disease, Framingham risk score, medication use (adjusted OR 1.8, 95%CI 1.1–3.0, p = 0.03). No beneficial effect on FMD was seen for those who drank more than 2 drinks/day.

**Conclusion:**

In conclusion, consumption of up to 2 alcoholic beverages per day was independently associated with better FMD compared to no alcohol consumption in this multiethnic population. This effect on FMD may represent an important mechanism in explaining the protective effect of alcohol intake on cardiovascular disease.

## Background

Epidemiologic evidence suggests that moderate alcohol consumption is associated with a reduced risk of developing coronary artery disease (CAD) [1-4]. The relationship between alcohol intake and the risk of developing CAD is a J- or U-shaped dose-response: the risk is higher in individuals not consuming any alcohol, low when alcohol consumption is low or moderate, and tends to go up again when alcohol consumption is high[5]. The mechanism underlying the cardioprotective effects of moderate alcohol consumption may involve prevention of atherogenesis through beneficial alcohol-induced changes in serum lipids, blood clotting proteins and insulin resistance[5], although not all of these changes are well understood. Endothelial dysfunction contributes to atherosclerosis and to the pathogenesis of cardiovascular disease[6,7], but few human studies have examined the effects of alcohol on endothelial function. Flow-mediated dilation (FMD) of the brachial artery assessed by high-resolution ultrasonography is widely used for the non-invasive assessment of endothelial function[8]. FMD is diminished in patients with vascular risk factors such as aging, smoking, hypertension, dyslipidemia and diabetes mellitus, and FMD improves with risk-reduction therapy[6,7]. The objective of this study was to investigate the association between alcohol consumption and endothelial function in order to explore one potential mechanism of the protective effect of alcohol intake on cardiovascular disease.

## Methods

### Study population

Subjects were drawn from the Northern Manhattan Study (NOMAS), an ongoing population-based prospective cohort study designed to investigate cardiovascular and stroke risk factors in a multi-ethnic sample (white, black, and Hispanic) of stroke-free individuals from northern Manhattan. Recruitment modalities have been previously published[9,10]. Briefly, between January 1998 and April 2001, high quality ultrasound images and measurements of FMD were performed in 884 participants from NOMAS participants. Written informed consent was obtained from all subjects, and the study was approved by the Institutional Review Board of Columbia University Medical Center.

### Baseline clinical evaluation

Baseline evaluation was performed at enrollment as previously reported[9,10]. Briefly, race-ethnicity was categorized into four groups: Hispanic, non-Hispanic black, non-Hispanic white, and other. Blood was taken from fasting subjects, and standard enzymatic methods were used for the sample analyses. Cardiovascular risk factors were collected by direct interview and were defined as in the prior publication[10]. Hypertension was defined as a systolic blood pressure (SBP) recording >140 mmHg or diastolic blood pressure (DBP) recording >90 mmHg during one sitting based on the mean of two readings of the blood pressure measurements, a patient's self-report of a history of hypertension, or antihypertensive medication use. Diabetes mellitus was defined by one or more of the following: a patient's self-report of such a history, insulin use, oral hypoglycemic use, or fasting glucose>126 mg/dl. Hypercholesterolemia was defined by a patient's self-report of hypercholesterolemia, use of lipid lowering therapy, or a fasting total cholesterol level >240 mg/dL. Body mass index was calculated as weight (kg) divided by height (m) squared. Coronary artery disease (CAD) was defined by self-report of history of heart attack, bypass surgery or angioplasty. Framingham risk score was computed from baseline risk factor data. This score takes into account data on age (per each 5 years), total cholesterol, high density lipoprotein cholesterol, systolic blood pressure, and cigarette smoking and estimate the 10-year risk for coronary artery disease (CAD) in each gender[11].

### Flow-mediated dilation assessment

Arterial endothelial function defined as the brachial artery response to hyperemia was non-invasively assessed using high resolution B-mode ultrasonography as previously described[10]. Participants fasted for 12 hours, and also avoided exercise for at least 4–6 hours prior to the FMD examinations. The brachial artery diameter was measured 6 cm above the antecubital crease using a 15 MHz liner array transducer (Phillips 5500, Andover, MA). FMD was measured as the dilator response to reactive hyperemia induced by 5 minute blood pressure cuff occlusion of the upper arm. The cuff was inflated to at least 50 mmHg above SBP to occlude arterial flow. End-diastolic images were acquired and digitized by a frame grabber (model LG3, Scion Corporation) at baseline and 1 min after cuff deflation. A blinded reader analyzed brachial artery diameters off-line using analysis software. Three consecutive cardiac cycles were analyzed for both baseline and hyperemia studies of each subject, and the measurements averaged. FMD was expressed as percent change from rest:

$$\text{FMD }=\frac{\text{brachial artery diameter at hyperemia - brachial artery diameter at baseline}}{\text{brachial artery diameterat baseline}}\times 100$$

Intraobserver variability for FMD measurements was 1.3%.

### Alcohol consumption assessment

The alcohol consumption assessment was described previously[12,13]. Research assistants used structured in-person interviews adapted from the National Cancer Institute Food Frequency questionnaire[14] and the Willett Food Frequency questionnaire[15]. Inquiries were made about consumption of wine, beer and liquor on average during each participant's lifetime. The defined response regarding frequency allowed 9 possibilities ranging from never to more than 6 drinks per day. The responses for each beverage type were summed to obtain a total quantity, and an average daily quantity was calculated. A standard drink of wine was considered to contain 4 ounces, beer 12 ounces, and liquor 1.5 ounces of ethanol. The reliability and validity of alcohol assessment has been shown previously to be good in our population[12].

### Statistical analysis

The categorization of alcohol consumption was the same as in the previous publications[13,1] less than 1 drink/month; [2] 1 drink/month to 2 drinks/day; and [3] more than 2 drinks/day. All statistical analyses were performed using SAS 9.1 (SAS Institute, Cary, NC). The association between FMD and alcohol consumption was analyzed by univariate and multivariable logistic regression analyses. The dependent variable, FMD, was dichotomized using the median level of the FMD distribution.

## Results

Table 1 shows the characteristics of the 884 participants. The mean age was 66.8 years, 43.4% were men. The mean brachial FMD was 5.7% and the median was 5.5%. Table 2 shows that moderate drinkers (1 drink/month to 2 drinks/day) were more likely to have FMD above the median of FMD (unadjusted OR 1.7, 95% CI 1.2–2.4, *p *= 0.005) compared to non-drinkers. In multivariate analysis, the relationship between moderate lifetime alcohol consumption and FMD remained significant after adjusting for sex, race-ethnicity, body mass index, diabetes mellitus, CAD, Framingham risk score and medication use (cholesterol lowering agents, aspirin, insulin, oral hypoglycemic agents, ACE inhibitors, beta-blockers, calcium channel blockers and diuretics) (adjusted OR 1.8, 95%CI 1.1–3.0 *p *= 0.03). No beneficial effect on FMD was seen for those who drank more than 2 drinks/day.

**Table 1:** Characteristics of study participants

Characteristic	Mean (SD) or n (%)
Total number	884
Age (years)	66.8 (8.8)
Men	384 (43.4)
Race-ethnicity	
Hispanics	596 (67.4)
Non-Hispanic Black	154 (17.4)
Non-Hispanic White	134 (15.2)
Hypertension	603 (68.2)
Diabetes Mellitus	202 (22.9)
Hyperlipidemia	435(49.3%)
Current smoking	140 (16.0)
History of smoking	478 (54.1)
Body mass index (kg/m^2^)	27.9 (5.1)
Systolic BP (mmHg)	143 (19)
Diastolic BP (mmHg)	84 (10)
Total cholesterol (mg/dl)	202.1 (39.7)
HDL cholesterol (mg/dl)	45.6 (14.0)
LDL cholesterol (mg/dl)	130.2 (35.8)
Blood Glucose (mg/dl)	105.0 (48.8)
CAD	74(8.4%)
Framingham risk score	16.9+/-3.7
FMD(%), mean(SD)	5.7 (3.8)
FMD(%), median	5.5
Medications	
Aspirin	244(27.7%)
Cholesterol lowering agents	162(18.4%)
Insulin	37(4.2%)
Oral hypoglycemic	129(14.7%)
Medication for blood pressure	393(69.8%)
ACE inhibitor	45(5.1%)
Beta-Blocker	41(4.7%)
Calcium channel blocker	40(4.6%)
Diuretics	124(14.1%)

**Table 2:** Odds Ratios for high FMD by categories of alcohol consumption

Average alcohol consumption		OR	unadjusted 95% CI	p	OR	adjusted 95% CI	p
< 1 drink/month	147	1			1		
1 drink/m to 2 drinks/d	618	1.69	1.17–2.44	0.005	1.78	1.07–2.99	0.03
> 2 drinks/day	119	1.56	0.96–2.54	N.S	1.59	0.80–3.17	N.S

Adjusted for sex, race-ethnicity, body mass index, diabetes mellitus, coronary artery disease, Framingham risk score, medication use.

Among the 884 study participants, 74 (8.4%) subjects had CAD. Among those 74 subjects, 18 (24.3%) had less than 1 drink/month, 44 (59.5%) had 1 drink/month to 2 drinks/day, and 12 (16.2%) had more than 2 drinks/day. Among the 810 subjects who did not have CAD, 129 (15.9%) had less than 1 drink/month, 574 (70.9%) had 1 drink/month to 2 drinks/day, and 107 (13.2%) had more than 2 drinks/day. No association was found between CAD and alcohol consumption (Chi-square test = 4.6 with 2 degrees of freedom, *p *= N.S).

## Discussion

Our data provide evidence that moderate alcohol consumption is associated with better FMD, independent of traditional vascular risk factors. The categorization of moderate alcohol consumption was the same as in a previous publication[13]. To the best of our knowledge, our study is the first to obtain data from a large multi-ethnic, population-based cohort showing the beneficial effect of moderate alcohol drinking on FMD. Distinctive features of our cohort are the elderly, mostly Hispanic population, a high proportion of women, high prevalence of hypertension, high body mass index, and lower than expected proportion of smokers. Other studies in selected populations have shown similar associations. For example, FMD was higher in moderate drinkers compared to non-drinkers among Japanese men with CAD[16].

Brachial artery FMD reflects NO-dependent endothelial function[17]. The synthesis of endothelial NO from L-arginine is regulated by endogenous nitric oxide synthase (eNOS)[18]. The endothelium regulates vascular homeostasis and is essential for vasodilation in response to increases in blood flow-associated shear stress[19]. Endothelial dysfunction is characterized by an impaired endothelium-dependent vasodilation response and decreased production and local bioavailability of NO[6]. Impaired FMD is also associated with atherosclerosis and predisposes to CAD.

Our findings indicate the possibility that a chronic effect of moderate alcohol consumption is beneficial to endothelial function. While the acute and chronic responses of vascular endothelium to alcohol may be different, neither is fully understood. In acute interventional studies, most of which assessed the effect of wine, some reported that FMD of the brachial artery was improved several hours after ingestion of wine[20], while others observed that the polyphenolic components, rather than alcohol itself, improved FMD among healthy volunteers and patients with CAD[21,22]. One study reported that alcohol produced significant vasodilatation of a brachial artery at resting condition and it led to a significant increase in the artery diameter at reactive hyperemia; however, the percentage of FMD did not change in healthy subjects[23].

Previous *in vitro *experimental studies suggested that low concentrations of alcohol promote endothelial cell survival[24] and increase eNOS expression and NO production in both basal and flow stimulated activity [25-27]. These findings might provide a mechanism to explain the possible beneficial effect of moderate alcohol consumption on endothelial function.

Several putative biological mechanisms have been proposed to explain the cardioprotective effect of moderate alcohol consumption. Moderate alcohol consumption increases HDL, reduces platelet aggregation and fibrinogen levels, increases fibrinolysis, and also improves blood insulin sensitivity and reduces insulin resistance[5]. Although we were not able to adjust for all of these factors, our data suggest the possibility that moderate alcohol consumption directly influences endothelial function. Further research would be necessary to assess the relationship between alcohol and endothelial function both in acute and long term human research and experimental research.

This study has several limitations. First, this is a cross-sectional study, and therefore a causal relationship cannot be established. Second, the use of self-reported information on alcohol intake may have introduced misclassification of exposure, most likely an underestimation of alcohol consumption. Third, we are not able to investigate any differential effect across types of alcoholic beverages. Fourth, due to unequal distribution of reported average alcohol consumption categories, there exists a possibility that this imbalance may have affected the results. Finally, we did not examine endothelial-independent vasodilation with nitroglycerin in our cohort.

## Conclusion

In conclusion, average consumption of up to 2 alcoholic beverages per day was independently associated with better FMD compared to consumption of less than 1 drink per month in this multiethnic population. This effect on FMD may represent an important mechanism in explaining the protective effect of alcohol consumption on cardiovascular disease.

## Competing interests

The authors declare that they have no competing interests.

## Authors' contributions

KS conceived of the study, participated in data analysis and interpretation and drafted the manuscript. MSVE participated in the design of the study, data analysis, interpretation of data, and drafting the manuscript. BB-A participated in the study coordination, data analysis and interpretation and in critical review of the manuscript. ZJ performed the statistical analysis Tand interpretation and critical review of the manuscript. GB participated in the analysis and interpretation of the data and drafting the manuscript. MRDT participated in obtaining funding for the study, the coordination of the study, analysis and interpretation of data and drafting the manuscript. RLS participated in obtaining funding for the study, the study design, data analysis and interpretation, and critically reviewed the manuscript. SH participated in the study design, data analysis and interpretation, and critically reviewed the manuscript. All authors read and approved the final manuscript.

## Pre-publication history

The pre-publication history for this paper can be accessed here:


